# Differentiation between Celiac Disease, Nonceliac Gluten Sensitivity, and Their Overlapping with Crohn's Disease: A Case Series

**DOI:** 10.1155/2013/248482

**Published:** 2013-01-27

**Authors:** Aristo Vojdani, David Perlmutter

**Affiliations:** ^1^Deptartment of Immunology, Immunosciences Lab., Inc., Los Angeles, CA 90035, USA; ^2^Cyrex Laboratories, Phoenix, AZ 85015, USA; ^3^Perlmutter Health Center, Naples, FL 34102, USA

## Abstract

Celiac disease (CD) and nonceliac gluten sensitivity (NCGS) are two distinct conditions triggered by the ingestion of gliadin. Although symptoms of nonceliac gluten sensitivity may resemble those of celiac disease, due to the lack of objective diagnostic tests, NCGS is associated with overlapping symptomatologies of autoimmunities and Crohn's disease. Furthermore, a gluten-free diet is only recommended for those who meet the criteria for a diagnosis of CD. Unfortunately, that leaves many nonceliac gluten-sensitive people suffering unnecessarily from very serious symptoms that put them at risk for complications of autoimmune disorders that might be resolved with a gluten-free diet. Thus, a new paradigm is needed for aid in diagnosing and distinguishing among various gut-related diseases, including CD, NCGS (also known as silent celiac disease), and gut-related autoimmunities. Herein, we report three different cases: the first, an elderly patient with celiac disease which was diagnosed based on signs and symptoms of malabsorption and by a proper lab test; second, a case of NCGS which was initially misdiagnosed as lupus but was detected as NCGS by a proper lab test with its associated autoimmunities, including gluten ataxia and neuromyelitis optica; third, a patient with NCGS overlapping with Crohn's disease. The symptomatologies of all three patients improved significantly after 12 months of gluten-free diet plus other modalities.

## 1. Introduction

Wheat allergy, celiac disease (CD), and nonceliac gluten sensitivity (NCGS) are three distinct conditions that are triggered by the ingestion of wheat gliadin [1–3]. In these conditions, the reaction to gluten is mediated by both cellular and humoral immune responses, resulting in the presentation of different symptomatologies. For example, in wheat allergy a specific sequence of gliadin peptides cross-links two IgE molecules on the surface of mast cells and basophils that trigger the release of mediators such as histamines and leukotrienes [4]. 

Celiac disease (CD) is an autoimmune condition with known genetic makeup and environmental triggers, such as gliadin peptides. CD affects between 1-2% of the general population.

Markers for confirming a diagnosis of this disorder are IgA against native, deamidated gliadin peptides, and IgA antitissue transglutaminase (tTg) autoantibody. In comparison with CD, nonceliac gluten sensitivity (NCGS) may affect from 6 to 7% of the population, [5–7]. According to two articles published in 2010 and 2011 by Sapone et al. [5, 6], symptoms in GS may resemble some of the gastrointestinal symptoms that are associated with CD or wheat allergy, but it is emphasized that objective diagnostic tests for nonceliac gluten sensitivity are currently missing [5, 6]. While studying the innate and immune responses in CD compared to those in NCGS, the researchers found that TLR1, TLR2, and TLR4, which are associated with innate immunity, were elevated in mucosal NCGS but not in CD, while biomarkers of adaptive immunity such as IFN-*γ*, IL-21, and IL-17A were expressed in mucosal tissue in CD but not NCGS. They believed that measurements of toll-like receptors and IFN-*γ*, IL-21, and IL-17A would enable them to differentiate between CD and NCGS [5, 6] with a method that is highly invasive and would require a biopsy. Immediate type 1 hypersensitivity to gluten is IgE mediated, while delayed type hypersensitivity to gluten is an antibody- (IgG, IgA) and T-cell-mediated reaction, which is called celiac disease or nonceliac gluten sensitivity with enteropathy [8]. In the absence of IgG and IgA against tTg, elevated IgG and IgA against various wheat antigens and peptides indicate the loss of mucosal immune tolerance against wheat peptides and the development of nonceliac gluten sensitivity [8]. Due to antigenic similarities between wheat antigens and human tissue, both CD and NCGS can result in many autoimmune conditions, including type 1 diabetes, arthritis, thyroiditis, and even neuroautoimmune conditions such as gluten ataxia and multiple sclerosis [9–11]. 

While NCGS patients, similar to CD patients, are unable to tolerate gluten and can develop the same or similar sets of gastrointestinal symptoms, in NCGS this immune reaction does not lead to small intestine damage [5, 6]. This lack of induction of intestinal damage in NCGS and the association of CD with genetic markers HLA DQ2/DQ8 plus small intestinal damage make the diagnosis of CD much easier than NCGS. The less severe clinical picture in NCGS, the absence of tTg autoantibodies, and the dismissal of the significance of elevated IgG and IgA autoantibodies against various wheat proteins and peptides by many clinicians make NCGS an extremely dangerous disorder. This is because the persistence of IgG and/or IgA antibodies in the blood for long periods of time along with inducers of inflammatory cascades can result in full-blown autoimmunity. If this were to be the case, due to the severity of the resulting tissue damage, even implementation of a gluten-free diet (GFD) might not be able to help reverse the course of the autoimmune reaction induced by IgG and IgA antibodies against different wheat antigens and peptides. 

Several studies have evaluated the possible neurological complications of CD, with emphasis on both the central nervous system and peripheral nervous system, particularly the involvement of small fiber neuropathy that seems to play a more important role in the pathogenesis of neurological complications of CD [9–16]. In some of these studies, the important finding was made that some patients who adopted a GFD and had CD in good remission still had an increased risk of clinical or subclinical neuropathy despite good adherence to the GFD [14]. These data reinforce the previous report of Volta et al., in which peripheral nervous disorders persisted in a 46-year-old female but improved significantly in a 38-year-old female despite a GFD [15]. This means that the duration of exposure to wheat antigens and antibody reactivity against the central and peripheral nervous systems, in particular cerebellar and ganglioside antibodies, plays a significant role in the recovery of patients from neurological manifestations of gluten reactivity after a GFD [10].

Similar results have been described for the association between CD and antineuronal antibodies. Their prevalence ranges from 22.22 to 61% in adults [10, 14], whereas in children the prevalence is about 5% [16]. The interesting finding of these reports is that in most cases these antibodies did not disappear after adoption of a GFD, except in a child reported by Briani et al. [16]. However, in their already mentioned study, Volta et al. [15] described the disappearance of antibodies within 1 year in most patients, as well as the regression of these antibodies in pediatric CD patients. Contrarily, in a more recent report [12], antineuronal antibodies did not disappear in any of the adult CD patients and in fact correlated with the persistence of neurological picture.

This raises the question: why does the GFD seem to work in some cases and not in others? Why do antineuronal antibodies disappear a year after implementation of a GFD in some patients and not in others? The first hypothesis is that 12 months may be sufficient for mucosal recovery but not for gluten-associated pathological conditions. But it is also possible that persistence of antibodies, as well as persistence of neurological symptoms, may be related to the duration of gluten exposure. There may be a first stage of neurological disease in CD when it is still gluten sensitive. During this stage a GFD may still result in both regression of neurological symptoms and the disappearance of antineuronal antibodies. The succeeding more advanced stage may, however, be considered gluten insensitive; in this phase both neurological symptoms and antineuronal antibodies persist despite a GFD, perhaps due to autoimmunity resulting from gluten. Thus, the key to explaining this apparent inconsistency in the efficacy of a GFD may be the duration of gluten ingestion [12].

Since, then, as in many autoimmune disorders the key seems to be the duration of exposure to the environmental triggers, in this case gluten exposure, our recommendation is to use the most sensitive biomarkers to diagnose CD or NCGS as early as possible, because in many adult patients delays in the diagnosis may cause severe and irreversible damage to various tissues, including the central and peripheral nervous systems [12, 17].

**Figure 1:**
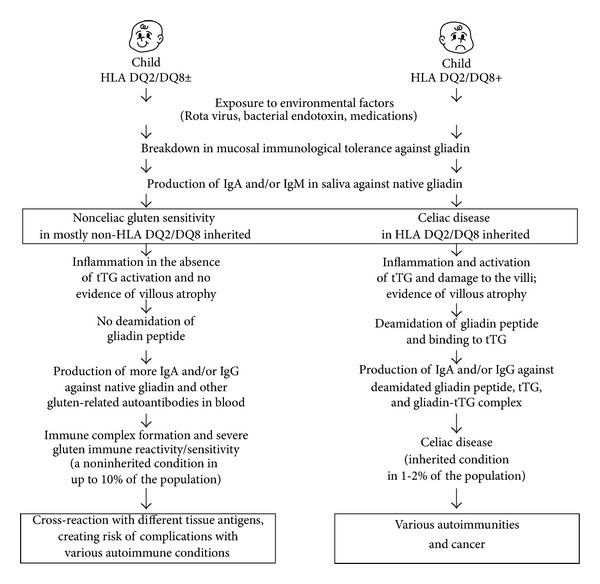
Differentiation between nonceliac gluten sensitivity and celiac disease.

A comparison between celiac disease and gluten immune reactivity/sensitivity is shown in Figure 1. According to this model, if two children, one with a negative genetic makeup (HLA DQ2/DQ8^−^), and the other with positive (HLA DQ2/DQ8^+^), are exposed to environmental factors, such as Rota virus, bacterial endotoxins, and some medications or their synergistic effects, the result can be a breakdown of mucosal immune tolerance in both children. The induction of mucosal immune tolerance against gliadin results in the production of IgA and/or IgG against native wheat proteins and peptides.

However, in the individual with the positive genetic makeup, the IgG and IgA antibodies against gliadin along with biomarkers of inflammation can activate tTg, induce damage to the villi, and result in villous atrophy. Deamidation of a specific gliadin peptide leads to the formation of a complex between it and the tTg; the presentation of this complex by antigen-presenting cells to T cells and B cells results in IgA or IgG production against tTg, deamidated gliadin, and the gliadin-tTg complex. The formation of these antibodies and their detection in blood is the hallmark of CD, which is an inherited condition detected in 1-2% of the population. If CD is left untreated, the outcome could be autoimmunities and cancer. 

In comparison, in an individual negative for HLA DQ2/DQ8, this breakdown in immunological tolerance and the concomitant production of IgA and or IgG against native wheat proteins and peptides may activate an inflammatory cascade. In the absence of tTg activation, however, villous atrophy does not occur. Furthermore, gliadin peptides do not go through deamidation, and consequently IgG and IgA antibodies are produced only against native wheat and gliadin peptides. 

With continuous exposure to wheat antigens and continuous mucosal immune tolerance, the wheat antigens and reacting antibodies form an unholy alliance of immune complexes, resulting in severe NCGS. This immune reactivity and sensitivity are a noninherited condition detected in up to 10% of the population. If this disorder is left unchecked, prolonged exposure to IgG and IgA antibodies against wheat antigens and peptides and their cross-reaction with different tissue antigens can result in various autoimmune disorders. 

## 2. Materials and Methods

The ELISA methodology for measuring antibodies against various wheat proteomes and tissue antigens has been described previously [17]. Briefly, the microwell plates were prepared and coated with the desired number of wheat-associated antigens and/or peptides. Calibrator and positive controls and diluted patient samples were added to the wells and autoantibodies recognizing different wheat antigens bound during the first incubation. After washing the wells to remove all unbound proteins, purified alkaline phosphatase-labeled rabbit anti-human IgG/IgA was added; unbound conjugate was then removed by a further wash step. 

Bound conjugate was visualized with paranitrophenyl phosphate (PNPP) substrate, which gives a yellow reaction product; the intensity of which is proportional to the concentration of autoantibody in the sample. Sodium hydroxide was added to each well to stop the reaction. The intensity of color was read at 405 nm.

## 3. Case Study Examples 

Three different case reports, the first on a patient with celiac disease, the second with nonceliac gluten sensitivity and autoimmunity, and the third with nonceliac gluten sensitivity overlapping with Crohn's disease are shown next.

### 3.1. Case Report No. 1: Diagnosis of Celiac Disease in the Elderly by the Use of IgA against Gliadin and Tissue Transglutaminase with Improvement on a Gluten-Free Diet

A 76-year-old man with longstanding dyspepsia, indigestion, tiredness, and rapid weight loss was referred for gastrointestinal evaluation. Blood tests showed macrocytic anemia with low concentrations of folate and vitamin B-12. The patient's hemoglobin concentration was 7.9 g/dL, albumin 32 g/L, and transglutaminase 212 mg/mL (normal range = 0–10 mg/mL). An urgent colonoscopy and duodenal biopsy were performed, which yielded macroscopically normal results. At this level his IgG and IgA concentrations against gliadin and transglutaminase were checked using FDA-approved kits. Both IgG and IgA against *α*-gliadin were very high; against transglutaminase, IgA but not IgG was 3.8-fold higher than the reference range. In view of the IgA positivity against gliadin and transglutaminase and diagnosis of celiac disease he was transfused with 2 units of packed cells and started on both a gluten-free diet and 20 mg of prednisone daily. Six months later he had gained about 12 pounds and showed few GI symptoms. Because of this improvement the patient became committed to the GFD. One year after the first performance of IgG and IgA antibody testing against gliadin and transglutaminase the repeat tests for these antibodies were negative, which is a further indication that disease management plus a GFD was instrumental in the treatment of this elderly patient with silent celiac disease. 

#### 3.1.1. Discussion

According to Catassi et al. [1, 18], celiac disease (CD) is one of the most common lifelong disorders in western countries. However, most cases of CD remain undiagnosed mostly due to the poor awareness of the primary care physician regarding this important affliction. Celiac disease is perceived as presenting GI symptoms accompanied by malabsorption. But many patients with celiac disease do not present GI symptoms. These individuals may have silent or atypical celiac disease, and the condition may present with iron deficiency, anemia, increased liver enzymes, osteoporosis, or neurological symptoms [19]. As used herein, the term “atypical celiac disease” refers to celiac disease in patients who have only subtle symptoms, and the term “silent celiac disease” refers to celiac disease in patients who are asymptomatic. 

The increasing recognition of celiac disease is attributed to the use of new serological assays with higher sensitivity and specificity. Until recently celiac disease was incorrectly perceived as being uncommon and detected mainly during infancy or childhood. However, it is now recognized that most cases of CD occur in adults 40–60 years old. Patients in this age group may present their symptoms, lab test results, and other examination signs in atypical fashion. In fact, according to a very recent publication, less than one in seven patients is correctly diagnosed with CD [20]. 

Consequently, as this case shows, if an adult patient presents with symptoms and signs suggesting malabsorption, testing for IgA antibody against gliadin and transglutaminase should be considered. If the test results are positive, celiac diseases should then be made a part of the differential diagnosis, based on which a gluten-free diet should be recommended. If the gluten-free diet should produce an improvement in symptoms, the patient should commit to the diet regardless of age. 

### 3.2. Case Report No. 2: A Patient with Nonceliac Gluten Sensitivity and Autoimmunity

Here, a case report is described in which the original presentation led to an erroneous diagnosis of irritable bowel syndrome, resulting in incorrect medical intervention. The correct diagnosis of nonceliac gluten sensitivity (NCGS) was made after years of mistreatment. A 49-year-old woman with abdominal pain, constipation, acid reflux, and headache was examined by an internist. Investigation revealed normal CBC with hemoglobin of 10.8 g/dL and normal chemistry profile including liver enzyme. Over several visits detailed biochemical and immunological profiles including ANA, rheumatoid factor, T3, T4, and TSH levels were performed, all testing within the normal range. After repeated complaints about GI discomfort, the patient was referred for GI evaluation. Both endoscopy and *H. pylori* test results were normal. The patient was diagnosed with irritable bowel syndrome and put on *β*-blockers and esomeprazole magnesium, which moderately improved her symptomatologies. Four years later, however, in addition to the old GI symptoms and headache, she presented symptoms of malaise, blurred vision, and facial rash. She was intermittently sleepy and irritable and experienced breathing problems. Further lab tests revealed that her hemoglobin was 9.7 g/dL with MCV of 72 fL, a raised erythrocyte sedimentation rate at 46 mm/1st hour (normal range 0–20 mm/1st hour), ANA of 1 : 80 (normal range < 40), mild elevation in IgA smooth muscle antibody, double-stranded DNA, and extractable nuclear antibodies were negative. Based on the available evidence, a diagnosis of systemic lupus erythematosus (SLE) was made by a rheumatologist, and treatment with steroids was commenced. There was some improvement in her overall state but her hemoglobin level continued to be low, while her ESR fluctuated. Two years later she developed difficulty in passing urine accompanied by tingling and sensory disturbance in her trunk and legs, which led to her being referred to a neurologist. The patient reported a band-like sensation in the trunk and reduced visual acuity (8/46 in the right eye, 8/23 in the left eye) with minimal eye pain but normal eye movement. Lab investigation revealed low hemoglobin, abnormal MCV, and low serum ferritin at 14 mg/L (normal range 10–150 mg/L), which confirmed iron deficiency. MRI scan of the brain showed extensive white matter abnormalities not typical of multiple sclerosis, and no abnormalities were detected in CSF examination. Blood and CSF examination showed no evidence of bacterial and viral infection including syphilis, mycobacteria, borrelia, EBV, CMV, HTLV, and herpes type-6. Visual evoked potentials showed delay in both optic nerves. In view of these abnormalities, and since tests for nonceliac gluten sensitivity had not been performed during the earlier investigations, the possibility of nonceliac gluten sensitivity was considered. A comprehensive IgG and IgA panel was ordered against a repertoire of wheat proteins and peptides, as well as against tTg and various tissue antigens. This comprehensive nonceliac gluten sensitivity and immune reactivity screen revealed IgG against wheat antigens, *α*-gliadin 33 and 17 mer, *γ*- and *ω*-gliadin, glutenin, gluteomorphin, prodynorphin, gliadin-tTg complex, wheat germ agglutinin, and glutamic acid decarboxylase 65 (GAD-65). IgA antibodies were detected against wheat antigens and wheat germ agglutinin (see Table 1). Interestingly, both IgG and in particular IgA tested against tTg were within the normal range.

Furthermore, antibodies against ganglioside, cerebellar, synapsin, myelin basic protein, collagen, thyroglobulin, thyroid peroxidase, and aquaporin-4 were tested, and all were 2–4 fold above the reference range. Upper GI endoscopy and biopsy revealed normal histology and intraepithelial lymphocytes. Overall the patient was diagnosed as having nonceliac gluten sensitivity with its associated autoimmunities, including gluten ataxia, headache, white matter abnormalities, and neuromyelitis optica. A five-day course of intravenous methylprednisolone was implemented, and gradually the sensory, motor, and visual symptoms improved. In addition, based on the very high levels of IgG and some IgA antibodies against a repertoire of wheat antigens and peptides, a gluten-free diet was introduced, and 12 weeks later marked improvement was observed in the patient's clinical symptomatology. She continued the 100% gluten-free diet under the observation of a dietitian, and the steroid treatment was stopped. Six months after introduction of the diet antibody tests against wheat antigens, peptides, and human tissue were repeated; more than 60% reduction in some antibody levels was observed, and the patient became almost asymptomatic. 

#### 3.2.1. Discussion

From this data we concluded that a patient may suffer from NCGS without having abnormal tissue histology or flat erosive gastritis and antibody against tTg based on which a diagnosis of celiac disease is normally made. If patients with NCGS are not detected in time based on the proper lab tests, in particular IgG and IgA antibodies against a repertoire of wheat proteins and peptides, patients' symptomatologies may mislead many clinicians into treating their patients for lupus, MS-like syndrome, neuromyelitis optica, and many other autoimmune disorders. Therefore, measurement of IgG and IgA antibodies against a repertoire of wheat antigens, peptides, and neuronal antigens is recommended for patients with signs and symptoms of autoimmunities so that intervention with a gluten-free diet will be instrumental in reversing the autoimmune conditions associated with NCGS. Otherwise, untreated and/or mistreated, the patient may develop multiple autoimmune disorders.

### 3.3. Case Report No. 3: A Patient with Nonceliac Gluten Sensitivity Overlapping with Crohn's Disease

Crohn's disease is an inflammatory disorder that often emerges during the second or third decade of life, affecting the terminal ileum in more than two-thirds of patients [21]. A combination of genetic and environmental factors, including a shift in gut microbiota and dysfunctional responses against them, is believed to lead to dysregulated immunity, altered intestinal barrier function, and possibly autoimmunity [22]. 

A 32-year-old man presented with gastrointestinal discomfort and diarrhea 2-3 times per month. Laboratory results including chemistry panel, CBC, iron, ferritin, transferrin, vitamin B-12, thyroid function, and urine analysis were within the median level of the normal range. Upon the second visit and continuation of GI symptoms he was referred to a GI specialist who ordered additional lab examinations, including microbiological evaluation of the stool and blood tests for antibodies against *H. pylori*, *Saccharomyces*, and gliadin. Stool testing for *Salmonella*, *Shigella*, *Yersinia*, *Campylobacter*, enteropathogenic and enterohemorrhagic *E. coli, *or* Clostridium difficile* came out negative. Regarding antibody examinations in the blood, IgG against *H. pylori* and IgA against Saccharomyces and gliadin were negative, but IgG against gliadin was moderately elevated at 59 U/mL (normal value ≤20 U/mL). The IgG antibody elevations were considered nonspecific or protective, and the patient was put on painkillers and sent home with no diagnosis of any specific disorder.

Three years later after seeing the frequency of the watery diarrhea increase to 3–5 times daily and losing 12 pounds of his body weight in the last two months, the patient went to another GI specialist for a second opinion. Gastric and duodenal biopsies were performed. While the endoscopy of the upper GI tract revealed gastritis of the antrum, histologically, gastric and duodenal biopsy turned out to be negative. D-xylose absorption test was performed; the resulting value of 1.89 g/5 h in urine was suggestive of malabsorption. Immunoserologically ANA titers were below 1 : 40, p-ANCA and c-ANCA were negative, but the IgA anti-*Saccharomyces* antigen (ASCA) was positive at 85 U/mL (normal ≤ 10 U/mL). Based on the increased frequency of watery diarrhea, abnormal D-xylose absorption, and positive IgA anti-ASCA, the diagnosis of Crohn's disease was made. A therapeutical trial using cholestyramine was initiated, but the frequency of the diarrhea remained unchanged. In addition the patient was treated with 230 mg of methylprednisolone and 2 × 1000 mg of mesalazine. Two years after this treatment the patient developed enteroenteric fistulae in the terminal ileum with sigmoid affection. After admission to the hospital, ileocolectomy was performed, and 22 cm of the ileum was resected. Upon his release remission maintenance with 3 × 500 mg of mesalazine was implemented. 

For eight years following this treatment the patient continued to suffer from increasing frequency of watery diarrhea and lost an additional 14 pounds. During this period several additional treatment attempts were made using aspirin, loperamide, and budesonide, unfortunately without significant clinical improvement. Furthermore, the patient was losing more weight on a monthly basis. A complete review of the medical history revealed the fact that almost thirteen years earlier, gliadin IgG antibody had been found to be elevated, which was considered normal at the time. Since all classical treatments for Crohn's disease had failed to improve the clinical picture over all the years, a comprehensive test for the assessment of gluten immune reactivity and sensitivity was ordered. This included IgG and IgA against wheat, native, and deamidated *α*-gliadin peptides, *γ*-gliadin, *ω*-gliadin, glutenin, gluteomorphin, prodynorphin, gliadin-tTg complex, transglutaminase, wheat germ agglutinin, and GAD-65. 

Results depicted in Table 2 show that the patient had a significant elevation of IgG antibodies against 11 out of 12 tested antigens, and IgA antibodies against wheat, *α*-gliadin 33 mer, *ω*-gliadin, prodynorphin, wheat germ agglutinin, and GAD-65 were detected at 2–5 fold above the normal range. Based on these results, in addition to Crohn's disease a diagnosis of nonceliac gluten sensitivity was also made. A diet consisting of rice, potato, and other gluten-free/yeast-free foods was commenced immediately, which led after six weeks to a complete cessation of diarrhea. Upon continuation of the gluten-free diet, not only did stool consistency become normal, but the patient also started gaining weight. On followup one year later the patient was back to a normal state and had regained more than 80% of his lost weight. 

#### 3.3.1. Discussion

This case demonstrates the association of Crohn's disease with nonceliac gluten sensitivity but not with celiac disease. Based on the impressive clinical response to the gluten-free diet plus the detection of IgG and IgA antibodies against various wheat antigens, and upon re-evaluation of the IgG antibody level detected 14 years earlier, the diagnosis of Crohn's disease with secondary malabsorption and NCGS was finally established. Since IgG antibodies against gliadin but not transglutaminase were detected, it can be argued that in this patient the disease was initiated with nonceliac gluten sensitivity and not Crohn's disease. 

It is contemplated herein that continuous exposure to environmental factors, such as wheat antigen-induced inflammation for a prolonged period of time, may result in inflammatory bowel disease or Crohn's disease.

## 4. Conclusions Regarding Case Reports

The case studies presented demonstrate the importance of expanding the understanding of the etiology and pathophysiology of the autoimmune disorders described and highlight the utility of novel laboratory evaluations described herein.

## Figures and Tables

**Table 1:** IgG and IgA antibody patterns of a patient with gluten immune reactivity/sensitivity/autoimmunity reacting against various wheat antigens, peptides, and tissue antigens expressed as optical density with calculation of indices.

	Wheat antigens	Alpha gliadin 33 mer	Alpha gliadin 17 mer	Gamma Gliadin 15 mer	Omega Gliadin 17 mer	Glutenin 21 mer	Gluteomorphin	Prodynorphin	Gliadin- tTg complex	∗tTg	Wheat germ agglutinin	∗∗GAD-65
						IgG						

Cal 1	0.33	0.42	0.34	0.33	0.38	0.38	0.36	0.35	0.33	0.35	0.35	0.36
Cal 2	0.36	0.48	0.36	0.38	0.42	0.44	0.39	0.40	0.45	0.38	0.38	0.43
	3.85	2.44	2.38	3.86	2.41	2.50	3.86	2.49	1.49	1.00	3.86	3.88
(OD)	3.85	2.36	2.33	3.86	2.40	2.40	3.86	2.40	1.58	0.96	3.86	3.87
Index	**11.10**	**5.33**	**6.81**	**10.79**	**6.05**	**5.99**	**10.23**	**6.60**	**3.91**	**2.69**	**10.59**	**9.79**
Ref. range	1.3	1.4	1.5	1.5	1.6	1.5	1.5	1.7	1.6	1.4	1.5	1.3

						IgA						

Cal 1	0.392	0.462	0.401	0.368	0.400	0.449	0.467	0.445	0.400	0.430	0.485	0.397
Cal 2	0.421	0.462	0.414	0.379	0.418	0.453	0.479	0.481	0.392	0.402	0.455	0.404
	3.862	0.429	0.350	0.208	0.364	0.269	0.401	0.336	0.335	0.438	3.868	0.359
(OD)	3.828	0.426	0.397	0.245	0.325	0.290	0.546	0.372	0.360	0.473	3.833	0.438
Index	**9.459**	**0.925**	**0.917**	**0.606**	**0.842**	**0.620**	**1.001**	**0.765**	**0.878**	**1.095**	**8.193**	**0.995**
Ref. range	2.4	1.8	2.0	1.9	1.8	1.7	1.8	1.8	1.6	1.5	1.9	1.5

*Transglutaminase.

∗∗Glutamic acid decarboxylase.

Index = mean OD of patients/mean OD of calibrators.

**Table 2:** IgG and IgA antibody patterns of a Patient with crohn's disease reacting against various wheat antigens, peptides, and tissue antigens expressed as optical density with calculation of indices.

	Wheat antigens	Alpha gliadin 33 mer	Alpha gliadin 17 mer	Gamma gliadin 15 mer	Omega gliadin 17 mer	Glutenin 21 mer	Gluteomorphin	Prodynorphin	Gliadin- tTg complex	∗tTg	Wheat germ agglutinin	∗∗GAD-65
IgG												

Cal 1	0.45	0.41	0.38	0.39	0.36	0.39	0.55	0.47	0.71	0.49	0.41	0.55
Cal 2	0.38	0.33	0.44	0.44	0.37	0.40	0.54	0.56	0.60	0.49	0.48	0.46
	3.86	3.79	3.86	3.67	3.85	3.24	3.84	3.86	1.71	3.80	3.82	3.84
(OD)	3.84	3.79	3.84	3.59	3.85	3.25	3.83	3.83	1.73	3.74	3.80	3.59
Index	**9.31**	**10.23**	**9.33**	**8.67**	**10.51**	**8.22**	**7.03**	**7.43**	**2.62**	**7.71**	**8.60**	**7.34**
Ref. range	1.3	1.4	1.5	1.5	1.6	1.5	1.5	1.7	1.6	1.4	1.5	1.3

IgA												

Cal 1	0.37	0.39	0.40	0.39	0.43	0.43	0.46	0.47	0.41	0.42	0.48	0.42
Cal 2	0.45	0.44	0.46	0.42	0.47	0.51	0.54	0.50	0.48	0.48	0.49	0.47
	3.89	3.56	0.99	0.99	2.25	1.05	1.12	3.85	1.00	1.18	3.73	3.87
(OD)	3.89	3.48	0.99	0.99	2.25	1.03	1.10	3.82	0.99	1.20	3.83	3.85
Index	**9.48**	**8.43**	**2.27**	**2.45**	**4.98**	**2.20**	**2.23**	**7.90**	**2.25**	**2.65**	**7.79**	**8.69**
Ref. range	2.4	1.8	2.0	1.9	1.8	1.7	1.8	1.8	1.6	1.5	1.9	1.5

*Transglutaminase.

∗∗Glutamic acid decarboxylase.

Index = mean OD of patients*⁄*mean OD of calibrators.
